# New retinoblastoma (RB) drug delivery approaches: anti‐tumor effect of atrial natriuretic peptide (ANP)‐conjugated hyaluronic‐acid‐coated gold nanoparticles for intraocular treatment of chemoresistant RB

**DOI:** 10.1002/1878-0261.13587

**Published:** 2024-01-12

**Authors:** André Haase, Natalia Miroschnikov, Stefan Klein, Annika Doege, Nicole Dünker, Dario Van Meenen, Andreas Junker, Achim Göpferich, Paola Stephanie Apaolaza, Maike Anna Busch

**Affiliations:** ^1^ Department of Neuroanatomy, Center for Translational Neuro‐ and Behavioral Sciences (C‐TNBS), Institute for Anatomy II University of Duisburg‐Essen, Medical Faculty Germany; ^2^ Institute of Neuropathology University of Duisburg‐Essen, Medical Faculty Germany; ^3^ Department of Pharmaceutical Technology University of Regensburg Germany; ^4^ Type 1 Diabetes Pathology Research Unit, Institute of Diabetes Research Helmholtz Centre Munich Germany

**Keywords:** ANP, CAM, chemoresistance, gold nanoparticles, hyaluronic acid, retinoblastoma

## Abstract

Intraocular drug delivery is a promising approach for treatment of ocular diseases. Chemotherapeutic drugs used in retinoblastoma (RB) treatment often lead to side effects and drug resistances. Therefore, new adjuvant therapies are needed to treat chemoresistant RBs. Biocompatible gold nanoparticles (GNPs) have unique antiangiogenic properties and can inhibit cancer progression. The combination of gold and low‐molecular‐weight hyaluronan (HA) enhances the stability of GNPs and promotes the distribution across ocular barriers. Attached to HA‐GNPs, the atrial natriuretic peptide (ANP), which diminishes neovascularization in the eye, is a promising new therapeutic agent for RB treatment. In the study presented, we established ANP‐coupled HA‐GNPs and investigated their effect on the tumor formation potential of chemoresistant RB cells in an *in ovo* chicken chorioallantoic membrane model and an orthotopic *in vivo* RB rat eye model. Treatment of etoposide‐resistant RB cells with ANP‐HA‐GNPs *in ovo* resulted in significantly reduced tumor growth and angiogenesis compared with controls. The antitumorigenic effect could be verified in the rat eye model, including a noninvasive application form via eye drops. Our data suggest that ANP‐HA‐GNPs represent a new minimally invasive, adjuvant treatment option for RB.

## Graphical abstract

The authors investigated new intraocular drug delivery approaches for adjuvant treatment of chemoresistant retinoblastoma (RB). Biocompatible gold nanoparticles (GNPs) were coated with hyaluronic acid (HA) and coupled to atrial natriuretic peptide (ANP). Treatment of chemotherapy‐resistant RB cells with ANP‐HA‐GNPs *in ovo* and in a rat eye model resulted in significantly reduced tumor growth, even after noninvasive application via eye drops.

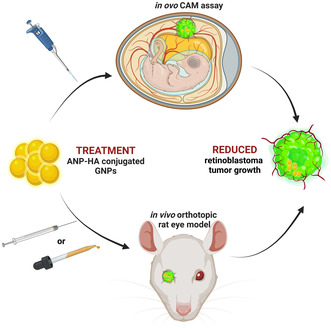

**Abbreviations**
ANP: atrial natriuretic peptide
ARPE‐19: adult retinal pigment epithelial cell line
CAM: chorioallantoic membrane
CO_2_
: carbon dioxide
CRX: cone‐rod homeobox
DAB: diaminobenzidin
DMEM: Dulbecco's modified Eagle medium
DNA: deoxyribonucleic acid
EDD: embryonic developmental day
etop: etoposide‐resistant
FBS: fetal bovine serum
GFP: green fluorescent protein
GMP: guanosine monophosphate
GNP: gold nanoparticles
HA: hyaluronic acid
HA‐SH: thiol‐functionalized hyaluronic acid
HEK293T: human embryonic kidney cells
IHC: immunohistochemistry
IL‐1β: interleukin‐1β
LDV: laser Doppler velocimetry
NPRA: natriuretic peptide receptor A
NPRC: natriuretic peptide receptor C
NVG: neovascular glaucoma
PBS: phosphate‐buffered saline
PCS: photon correlation spectroscopy
PDI: polydispersity index
PDR: proliferative diabetic retinopathy
PFA: paraformaldehyde
RB: retinoblastoma
RB1: retinoblastoma gene 1
RPE: retinal pigment epithelium cells
SEM: standard error of the mean
STR: short tandem repeat
TEM: transmission electron microscopy
TS: treatment setting
VEC: vincristine, etoposide, and carboplatin
VEGF: vascular epithelial growth factor
VEGFR2: vascular epithelial growth factor receptor 2
WERI: WERI‐Rb1

## 1 Introduction

Ocular drug delivery through noninvasive routes is challenging because of the anatomical structure and physiology of the eye, often resulting in low drug availability. Thus, for treatment of several retinal disorders, for example, proliferative diabetic retinopathy (PDR) [1], neovascular glaucoma (NVG) [2], and retinoblastoma intravitreal administration routes are currently used in order to reach the retina. Retinoblastoma (RB) is the predominant primary intraocular tumor found in children, occurring at a rate of 1 in 16 000 live births. It constitutes 2–4% of all childhood malignancies [3]. Retinoblastoma has an overall high survival rate, but further progressed tumors are often associated with high‐risk characteristics such as dissemination [4] and chemotherapy resistances. Initially, enucleation was the primary successful therapeutic approach for retinoblastoma. However, the emergence of new drug delivery routes, such as intra‐arterial, intravitreal, or intracameral, injections significantly increased ocular preservation rates and diminished the need for systemic chemotherapy [4, 5]. Chemotherapy with chemotherapeutics such as vincristine, etoposide, or carboplatin, which are routinely used in a combined RB VEC‐therapy often induce massive side effects and leads to drug resistances frequently limiting the treatment options of resistant tumors, which might cause relapses [6]. After therapy, 35% of retinoblastoma patients experience the development of secondary tumors, and among this group, 50% do not survive [7]. Emerging technologies, such as the utilization of nanoparticles as delivery systems for ocular drugs, small molecules, peptides, or nucleic acids, provide a noninvasive alternative treatment of retinoblastoma with increased accessibility, which is not only safe, but also long‐lasting.

For biomedical applications, plasmonic materials such as gold nanoparticles (GNPs) have unique advantages as antioxidant and antiangiogenic agents [8]. It has been shown that GNPs can reduce the proliferation and migration of retinal pigment epithelium cells (RPE) induced by vascular epithelial growth factor (VEGF) or interleukin IL‐1β [9]. Gold nanoparticles can be conjugated to different biomolecules [10] combing multiple advantages, including robust absorption and scattering of visible light, straightforward synthesis, manageable size and shape control, as well as high biocompatibility [11]. Following systemic administration, small‐sized gold nanoparticles can traverse physiological and anatomical ocular barriers, such as the blood‐retinal barrier. This expands the potential applications of GNPs as drug delivery systems through various administration routes [12]. We could recently show that a combination of a gold core with a hyaluronic acid (HA) coat is a promising candidate nanocarrier for treatment of eye diseases [11]. Hyaluronic acid is a FDA‐approved polymer commonly used in eye drops with the potential to enhance the delivery of anticancer drugs due to its CD44 receptor [13, 14]. Several cells of the eye express the CD44 receptor endogenously and in disease conditions, for example, retinal pigmented epithelium (RPE), Müller glial, and ganglion cells [13, 15]. Therefore, CD44 expressing cells have the capability to bind and internalize HA. Moreover, the composition of the ocular vitreous body contains not only 98% water and collagen but also hyaluronic acid (HA). The composition of this gel meshwork allows for a sustained release of drug molecules, extending the duration of their effect and enhancing bioavailability when administered in a solution [16]. Modifying the surface of GNPs with HA increases their mobility and permeability through ocular barriers, leading to antiangiogenic effects. This modification transforms GNPs into inhibitors of neovascularization [11]. In addition, HA enables larger nanoparticles to enter the cells as potential vehicles for the delivery of therapeutics to the posterior part of the eye via noninvasive application routes, for example, by eye drops [17].

The atrial natriuretic peptide (ANP), belonging to the family of atrial natriuretic peptides, plays an important role during stimulation of vasodilatation, natriuresis, and diuresis [18]. It has also been observed that ANP reduces choroidal neovascularization in the eye by inhibiting VEGF [19], likewise expressed in RB and correlated with increased RB malignancy [20]. Špiranec Spes et al. [21] recently demonstrated that ANP mitigates pathological retinal vascular regression and subsequent neovascularization through cyclic GMP signaling, protecting pericytes from apoptosis, and diminishing hypersecretion of VEGF from astrocytes. In addition, a combined treatment with glipizide, a second‐generation sulfonylurea hypoglycemic agent, and ANP suppressed breast cancer growth and metastasis by the inhibition of angiogenesis via the VEGF/VEGFR2 signaling pathway [22].

Against the background that HA improves ocular tissue distribution and inhibition of neovascularization by ANP potentially reduces tumor growth, the goal of the study presented was to evaluate the applicability of ANP‐conjugated, HA‐coated gold nanoparticles (ANP‐HA‐GNPs) as promising candidate nanocarriers for the treatment of chemoresistant RB tumors *in vivo*. For this purpose, we investigated potential suppressive effects of ANP‐HA‐GNPs on tumor growth and angiogenesis of aggressive etoposide‐resistant RB cell lines in an *in ovo* chorioallantoic membrane (CAM) model as well as in a newly established orthotopic *in vivo* RB rat eye model.

## 2 Materials and methods

### 2.1 Synthesis and characterization of ANP coupled hyaluronan‐modified gold nanoparticles

The synthesis of thiol‐modified HA and GNPs used in this manuscript was described previously [11]. Briefly, thiol‐functionalized HA (MW 5K; Lifecore™ Biomedical, Chaska, MN, USA) was cross‐linked by carboxylic acid mainly following the protocol previously described by Oliveira et al [23]. Gold nanoparticles were synthesized based on the reduction and stabilization of the salt form of gold (HAuCl_4_; Sigma‐Aldrich, Munich, Germany) by trisodium citrate dehydrate salt (Sigma‐Aldrich, Munich, Germany) by the Turkevich method [24]. The ratio of gold to citrate was 1 : 3.7 (w/w) to obtain GNPs with a 20 nm gold core. Thereafter, the ligand HA‐SH was covalently bound to the surface of gold by interaction of the thiol group and molecular gold as described previously [11].

The concentration of HA‐GNPs was determined by assessing the absorbance of the GNP core [25]. Gold nanoparticle surface modification with hyaluronic acid (HA) was identified by a shift in the maximum peak of the UV–visible spectrum. Measurements were conducted using a FLUOstar Omega microplate reader (BMG, Labtech, Ortenberg, Germany). The concentration of HA‐SH covalently bound to GNPs was indirectly determined using the Ellman's method.The ANP was then bound by electrostatic interaction to the HA component of the nanoparticle surface. Complete binding of the ANP to the HA‐GNPs was confirmed by SDS/PAGE electrophoresis (data not shown).

### 2.2 Size and zeta potential measurements

Particle size analysis of GNPs, HA‐GNPs, and ANP‐HA‐GNPs was conducted through photon correlation spectroscopy (PCS), while zeta potentials were measured using Laser Doppler Velocimetry (LDV). Both analyses were performed on a Zetasizer Nanoseries‐Nano ZS instrument (Malvern Instruments, Lappersdorf, Germany). All samples were appropriately diluted in Milli‐Q™ (Merck, Darmstadt, Germany) water for the analyses.

### 2.3 Transmission electron microscopy

Transmission electron microscopy (TEM) was conducted using a Libra 120 electron microscope (Carl Zeiss, Oberkochen, Germany). The procedures followed were consistent with protocols published previously [26, 27].

### 2.4 Cell lines and culture

The human retinoblastoma (RB) cell lines Y79 [28] (RRID: CVCL_1893) and WERI [29] (RRID: CVCL_1792), originally purchased from the Leibniz Institute DSMZ (German Collection of Microorganisms and Cell Cultures) were placed to our disposal by H. Stephan along with the RB cell line RB355 [30] (RRID: CVCL_S611), initially provided by K. Heise, and the corresponding etoposide‐resistant RB cell lines Y79‐Etop, WERI‐Etop, and RB355‐Etop. The cultivation protocols for these cell lines, as well as for human embryonic kidney cells (HEK293T, RRID: CVCL_0063) kindly provided by B. Royer‐Pokora and originally purchased from DSMZ, were comprehensively described in a prior publication [31].

All cell lines used were initially tested and authenticated by STR analysis. In the following, samples of all tested cells were frozen to insure access to tested cells in the course of all experiments. In addition, the RB cell lines were regularly analyzed for their individual *RB1* mutation status. The adult retinal pigment epithelial cell line ARPE‐19 (RRID:CVCL_0145) was purchased from ATCC (Manassas, VA, USA) and maintained in DMEM/F12 medium supplemented with 1% penicillin streptomycin (10 000 U·mL^−1^) and 10% inactivated FCS (GIBCO^®^, ThermoFisher Scientific, Darmstadt, Germany) at 37 °C in 5% CO_2_ atmosphere. No ethics approval was required for work with the human cell lines. All cell lines were tested for mycoplasms on a regular basis.

### 2.5 Intracellular distribution of nanoparticles

ARPE‐19 cells (65 000) were seeded on poly‐l‐lysine (0.1 mg·mL^−1^ for 30 min at 37 °C; GIBCO^®^, ThermoFisher Scientific) treated coverslips in a 24‐well plate and incubated overnight. Thereafter, 25 μm GNPs or ANP‐HA‐GNPs were added to the cells. Following a 24‐h incubation period, ARPE‐19 cells were fixed using paraformaldehyde (PFA; 4% in PBS; pH 7.4). Incorporated particles were stained using a silver staining kit (Sigma‐Aldrich, Munich, Germany) according to the manufacturer's protocol. Photomicrographs were captured using a Zeiss Axiovert 200 microscope (Zeiss, Jena, Germany).

### 2.6 *Ex vivo* vitreous humor diffusion and retinal explant studies

To get fresh vitreous humor and retinae, porcine eyes from a local slaughterhouse were enucleated after the animals were sacrificed. The eyes were transported in cold (4 °C) CO_2_‐independent l‐glutamine medium (GIBCO, ThermoFisher Scientific) until needed. The vitreous humor was separated from the retina and adjacent tissues and placed in a Petri‐dish (25 cm^2^; Corning, Kaiserslautern, Germany). To investigate the diffusion effect, 100 μL (0.5 mm) of ANP‐HA‐GNPs were injected into the vitreous body and monitored under a normal bright field camera (Nikon, Düsseldorf, Germany) for 24 h at room temperature.

In order to study the retinal distribution of ANP‐HA‐GNPs, conventional porcine retinal explants were dissected and cultured as described previously [32]. A trephine blade (ø 8 mm; Beaver Visitec, Waltham, MA, USA) was used to cut out a circular piece of retina from each eye with the inner limiting membrane (ILM) facing up. The retinal explants underwent treatment with 0.5 mm ANP‐HA‐GNPs. Neurobasal medium (GIBCO, ThermoFisher Scientific) was used for the dilution of the GNPs. Retinal cryosections (12 μm) were mounted on Superfrost Plus slides (ThermoFisher Scientific). Bright field microscopy visualization within the retinal tissue 24‐h postadministration of the particles was performed utilizing a Zeiss Axiovert 200 microscope (Zeiss).

### 2.7 Generation of lentiviral particles for stable transduction

For the transduction of WERI and RB355 RB cell lines, lentiviral particles were generated. For this pupose, 6 × 10^6^ human embryonic kidney cells (HEK293T) were transfected with 6 μg of each of the following plasmid DNAs: packaging vectors pczVSV‐G [33] and pCD NL‐BH [33], and pCL6LucEGwo (provided by H. Hanenberg). This transfection was carried out in DMEM medium in the presence of 45 μg polyethyleneimine (PEI, branched; Sigma‐Aldrich, St. Louis, MO, USA). The lentiviral vector (pCL6LucEGwo) contained a fusion of the human codon usage‐optimized luciferase (InvivoGen, San Diego, CA, USA) and an enhanced green fluorescent protein (EGFP; Clontech, Mountain View, CA, USA), driven by a modified spleen focus‐forming virus (SFFV) retrovirus U3 promoter [34]. Twenty‐four hours later, we exchanged the medium. After 72‐h cultivation in Iscove's Modified Dulbecco's medium (IMDM; Pan‐Biotech, Aidenbach, Germany) with 10% FBS and 1% penicillin/streptomycin, viral supernatants were harvested, filtered (0.45 μm filter), and cryoconserved. In order to stably transfect RB cells with luciferase/GFP for *in vivo* tumor formation experiments, cells were seeded in DMEM medium at a concentration of 1.25 × 10^6^. After 1 day, the medium was discarded and the WERI/WERI‐Etop or RB355/RB355‐Etop RB cells were transduced with the virus particles in the presence of polybrene (5 μL per ml lentivirus; H9268; Sigma‐Aldrich, München, Germany). Dulbecco's modified eagle medium with supplements (twice the volume of the virus particles) was added after another 24 h, and additional 48 h later, the medium was changed completely.

### 2.8 *In ovo* tumor formation

To assess alterations in tumor formation, etoposide‐resistant RB cells (Y79‐Etop and WERI‐Etop) and their respective control cells were grafted onto the chick chorioallantoic membrane (CAM) mainly following the protocol of Zijlstra and Palmer [35, 36] with modifications described previously [37]. Briefly, 1 × 10^6^ RB cells per egg were directly grafted onto the CAM membrane at embryonic developmental day 10 (EDD10) without using silicone rings. After 24 h, at EDD11, the grafted eggs were treated by dropping 40 μL solution containing particles (at a concentration of 1 mm) and/or ANP (at a concentration of 0.06 μg·μL^−1^) onto the CAM area, where the RB had previous been grafted. The different treatment conditions were as follows: (a) GNPs alone (1 mm stock), (b) HA‐GNPs (0.9 mm stock), (c) ANP‐HA‐GNPs (0.865 mm stock), (d) ANP alone (1 mg·mL^−1^ stock), or (e) PBS (control). Five to seven eggs were grafted in at least three independent experiments for each condition. Seven days after grafting and 6 days after treatment (EDD17), tumors that formed from the grafted cells were excised, measured, and photographed as described previously [31, 38, 39]. The eggs were cooled down on ice for at least 15 min prior to preparation, and the chicken embryos were decapitated directly after opening of the eggs. Vessel formation was analyzed with regard to total vessel area, vessel length, thickness, and branching points based on CAM tumors photographed *in situ* using the ikosa online software (KLM Vision, Graz, Austria).

### 2.9 *In vivo* orthotopic rat eye model

All experiments were approved by the state office for nature, environment, and consumer protection NRW (LANUV) under the reference numbers 81‐02.04.2018.A003 and 81‐02.04.2021.A015. The animals were anesthetized with isoflurane and euthanized by CO_2_. Sex‐independent Lewis rats (LEW/HanHsd) provided by the central animal laboratory of Essen (ZTL) were maintained on a 12‐h light–dark cycle with *ad libitum* access to food and water. Before anesthesia, rats were weighed, and metamizol (100 mg·kg^−1^) was administered orally for prophylactic pain prevention. Within the first 24 h of birth, newborn rat pups (P0) were anesthetized with isoflurane (3.0% isoflurane at an Ø flow of 1.5 L·min^−1^; Sigma Delta Vaporizer, Penlon, UK). The pups got an individual tattoo on their paws for identification during the experimental procedure and were transferred to a heated pad to ensure a body temperature of 37 °C. Maintenance of anesthesia (2.5% isoflurane at an O flow rate of 1.5 L·min^−1^) was assured using a nosecone for rat pups under a microscope. A 33 G needle attached to a 5‐μL Hamilton syringe was used bevel‐up to inject the RB cells through the naturally closed eyelid into the vitreous of the rats' eyes. Each animal received a single injection of 1 × 10^5^ luciferase‐GFP labeled WERI (*n* = 24), WERI‐Etop (*n* = 21), RB355 (*n* = 18), RB355‐Etop (*n* = 21) cells or an injection of sterile phosphate‐buffered saline (PBS control; *n* = 3) into the right eye. Injection of RB cells or PBS control (1 μL total volume for all injections) was done manually under a microscope. The RB cell suspension or PBS was injected slowly, and the needle remained in place for 30 s following injection to avoid reflux during extraction of the needle. The pups were returned to their mother in their home cage, and tumor formation was consecutively monitored by bioluminescence measurements over a time period of 9 weeks as described below. After 2 (P14), 4 (P28), or 9 weeks (P63), the rats were euthanized, and the right eye was removed and investigated immunhistochemically.

### 2.10 Bioluminescence imaging

Isoflurane anesthesia was performed as described above, following a subcutaneous injection of VivoGlo D‐luciferin potassium salt (Promega, Fitchburg, MA, USA) from a 40 mg·mL^−1^ stock in PBS. The dosage was calculated after weighting the animals prior to imaging (150 mg luciferin per kg animal weight). On the indicated days after injection (P3, P7, P10, P14, P28, P42), the anesthetized animals were imaged under maintenance of anesthesia using a Caliper Lumina II system (PerkinElmer, Waltham, MA, USA). Animals were imaged with their right side up, three to five at a time using full‐frame camera height. Images were gathered over 45 s each. Image visualization and quantification were performed using the living image analysis software 4.7.4 (PerkinElmer).

### 2.11 Treatment of etoposide‐resistant WERI cells with ANP‐HA‐GNPs in an orthotopic rat eye model

**Fig. 1:**
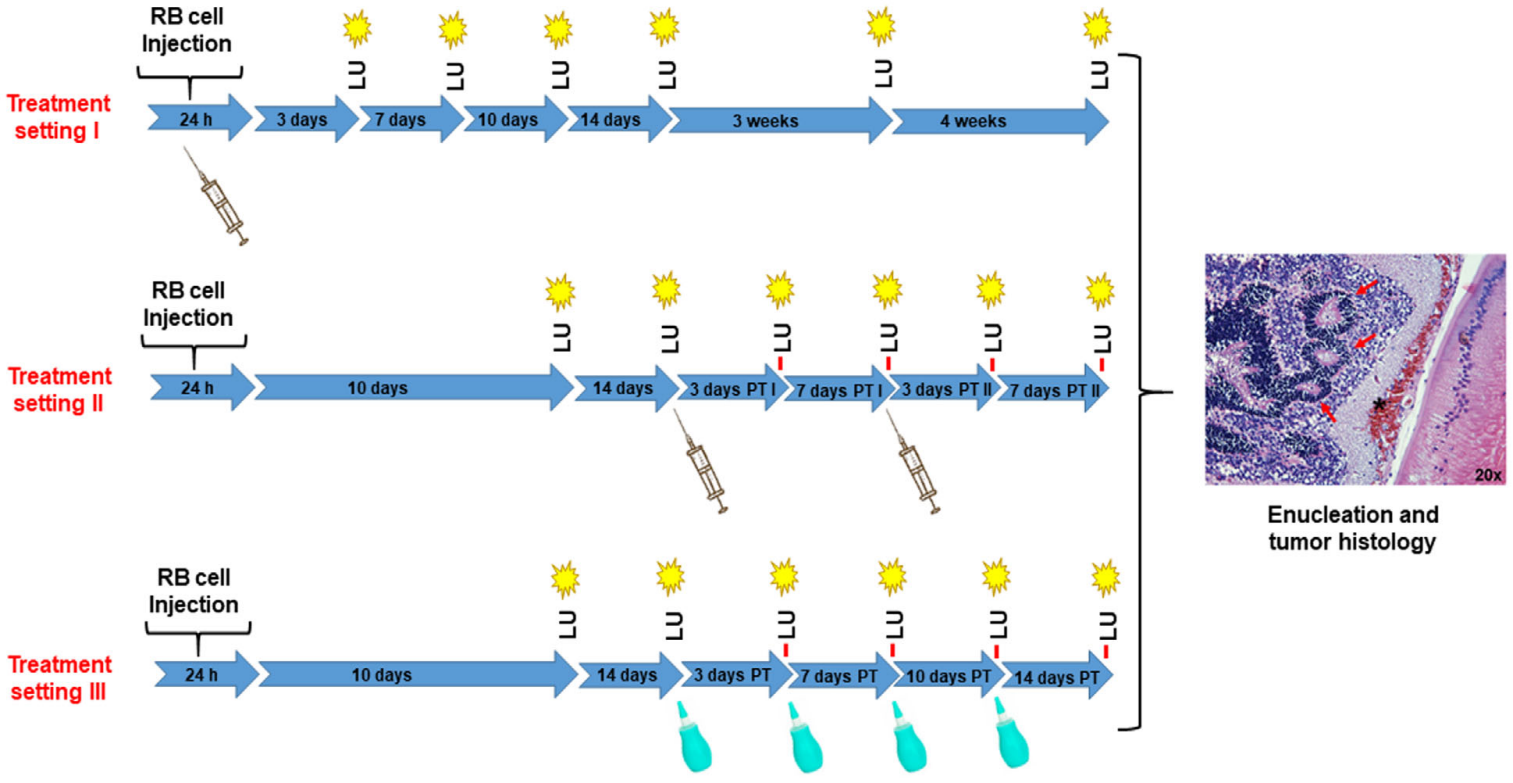
Workflow of retinoblastoma (RB) tumor cell injection into the naturally closed eye of newborn rats (P0) and the detection of luciferase signals under nanoparticle treatment. Treatment setting I: *n* = 12 treated and *n* = 10 control animals; Treatment setting II: *n* = 12 treated and *n* = 11 control animals; Treatment setting III: *n* = 12 treated and *n* = 12 control animals; LU, luminescence measurement; PT, post‐treatment; syringe: time point of nanoparticle treatment; green dropping bottle: time‐point of topical nanoparticle treatment; red arrows in the tumor histology demarcate rosette like RB tumor structures.

In order to investigate the effectiveness of a nanoparticle therapy on tumor development of etoposide resistant RB cells, three different treatment regimen (Fig. 1) were investigated based on the cell line WERI‐Etop labeled with luciferase and GFP (luc‐GFP). In treatment setting I, 1 × 10^5^ WERI‐Etop cells in 1 μL ANP‐HA‐GNPs solution (0.865 mm stock) were injected into the right eye of P0 rat pubs (Fig. 1). Animals were imaged on the indicated days after injection (P3, P7, P10, P14, P21, and P28) in comparison with control cells (WERI‐Etop) without treatment. In treatment setting II and III, RB tumors were allowed to grow 14 days prior to treatment. After 10 (P10) and 14 (P14) days, the animals were imaged. Those with detectable RB tumor growth were treated with 1 μL ANP‐HA‐GNPs solution (0.865 mm stock) via intravitreal injection on P14 and P21 in treatment setting II and with eye drops (1 μL ANP‐HA‐GNPs solution) on P14, P17, P21, and P24 in treatment setting III (Fig. 1). In treatment setting II and III, animals were imaged on the indicated days (P10, P14, P17, P21, P24, and P28) in comparison with control cells (WERI‐Etop) injected or dropped with PBS as vehicle under the same schedule. After 4 weeks (P28), rats were euthanized, and the right eye was enucleated and investigated immunohistochemically.

### 2.12 Histological and immunohistological processing

Upon completion of the experimental protocols, animals were euthanized by CO_2_ and eyes were enucleated. Whole P14, P28, and P63 eyes or CAM tumors were fixed in 4% paraformaldehyde overnight, dehydrated, paraffin‐embedded, and 5 μm sections were obtained using a microtome. Sections were deparaffinized and rehydrated in an ethanol series of descending concentration. Subsequently, sections were stained using Mayer's hematoxylin. Besides, immunohistochemical detection was performed using a ready‐to‐use rabbit monoclonal antibody against Ki67 (clone 30‐9; Roche Ventana, Basel, Switzerland) or CRX red (dilution 1 : 50; clone A‐9; Santa Cruz Biotechnology, Heidelberg, Germany) with the OptiView DAB IHC detection kit (Thermo Fisher, Darmstadt, Germany) for visualization. Images were captured using a slide scanner (Leica, Wetzlar, Germany) and subsequently analyzed by am aperio image scope Software (Leica).

### 2.13 Statistical analysis

Statistical analyses were performed using graphpad prism 9 (GraphPad Software, Boston, MA, USA). Data represent means ± SEM, and results were analyzed by a Student's *t*‐test and considered significantly different if *P*‐value < 0.05 (*), *P*‐value < 0.01 (**), or *P*‐value < 0.001 (***). To quantify Ki67‐positive cells, regions of interest were extracted from scanned slides and analyzed using imagej [40]. In a macro, hue, saturation and brightness were determined from the individual images and the threshold values for these parameters were adjusted to the needs of the “Analyze Particles” function in order to determine the percentage of Ki67‐positive cells. Quantification of CAM vessel formation was performed with the ikosa online software (KLM Vision, Graz, Austria; https://app.ikosa.ai/).

## 3 Results

### 3.1 Physical characterization of GNPs and ANP‐HA‐GNPs

**Fig. 2:**
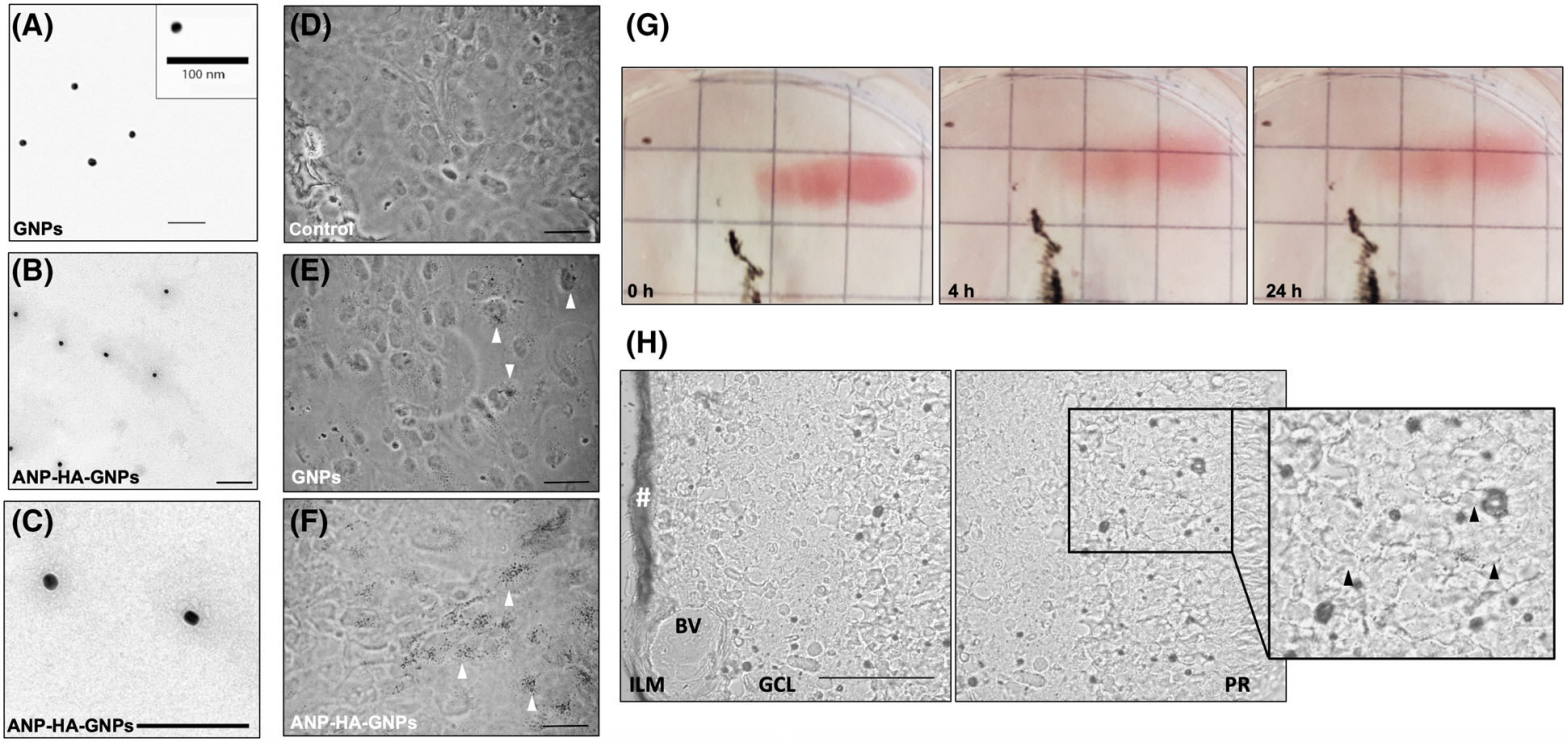
Characterization of atrial natriuretic peptide coupled hyaluronic acid coated gold nanoparticles (ANP‐HA‐GNPs). (A–C) Transmission electron microscopy (TEM) microscopy pictures of bare GNPs with 20 nm gold core (A) and modified ANP‐HA‐GNPs (B, C). Scale bars: 100 nm. (D–F) Bright field images of ARPE‐19 cells after silver staining for gold (black dots). Scale bars: 50 μm. Photographs were taken 24 h after treatment with 25 μm GNPs (E) or ANP‐HA‐GNPs (F) in comparison with nontreated control cells (D). White arrowheads exemplarily indicate some of the silver stained particles. (G) Photographs of porcine vitreous humor 0, 4 and 24 h after administration of 100 μL ANP‐HA‐GNPs. The black tissue represent remains of the retinal pigment epithelial layer. (H) Confocal bright field pictures of sequential cryosections of porcine retinal explants 24 h after administration of 0.5 mm ANP‐HA‐GNPs. Particles are visible as dotted black pattern within the tissues after silver staining. Accumulation/retention of ANP‐HA‐GNPs occurs at the administration side in the ILM and is marked by #. Black arrowheads indicate the location of the invaded particles. Scale bar: 50 μm. The experiments were performed in triplicates. ANP, atrial natriuretic peptide; ANP‐HA‐GNP, ANP coupled HA‐GNPs; BV, blood vessel; GCL, ganglion cell layer; GNP, gold nanoparticles; HA, hyaluronic acid; ILM, inner limiting membrane; PR, photoreceptor layer.

Table 1 displays the particle size, zeta potential, and polydispersity index (PDI) of the GNPs, both with and without attached hyaluronic acid (HA) or ANP‐HA. The attachment of HA or ANP‐HA to the GNP surface resulted in a significant increase in both, size and zeta potential, with values more than doubling. The PDI, reflecting the uniformity of particle size distribution, remained around 0.2 for bare 20 nm gold core GNPs (GNPs 20), HA‐GNPs, and ANP‐HA‐GNPs, indicating a homogeneous distribution. TEM microscopy analysis of the GNPs and ANP‐HA‐GNPs revealed that a polymer HA corona surrounded the ANP‐HA‐GNPs (Fig. 2B,C), whereas GNPs (Fig. 2A) lacked a visible corona. The presence of this surface layer accounted for the augmentation in size noticed in the PCS measurements (Table 1). To investigate the interactions of bare GNPs in comparison with ANP coupled HA‐coated GNPs (ANP‐HA‐GNPs), we analyzed the cellular nanoparticle uptake and distribution in ARPE‐19 cells (Fig. 2E,F) and nontreated cells as negative controls (Fig. 2D). Bare GNPs shown in Fig. 2E formed large vesicular aggregates inside the cells, whereas modified ANP‐HA‐GNPs displayed as small individual particles throughout the cytoplasm (Fig. 2F). As a proof of principle, we additionally analyzed the cellular uptake of ANP‐HA‐GNPs in RB cells and observed an internalization of the particles into WERI‐Etop cells (Fig. S1).

**Table 1:** Particle size, zeta potential, and polydispersity index (PDI) of the gold nanoparticles (GNPs); *n* ≥ 6; data represent mean ± standard deviation. ANP, atrial natriuretic peptide; ANP‐HA‐GNP, ANP coupled HA‐GNPs; HA‐GNP, hyaluronic acid coupled GNPs.

	SIZE (nm)	Zeta potential (mV)	PDI
GNPs	22.34 ± 0.54	−52.73 ± 7.30	0.292
HA‐GNPs	57.01 ± 3.86	−17.33 ± 0.67	0.224
ANP‐HA‐GNPs	59.26 ± 1.72	−7.58 ± 1.40	0.282

### 3.2 Vitreous humor diffusion and biodistribution in retinal explants

To analyze the diffusion of ANP‐HA‐GNPs into the vitreous humor, we utilized the visible red color of the GNPs as a qualitative indicator. The ANP‐HA‐GNP particles showed a good distribution in the vitreous humor after 4 and 24 h (Fig. 2G). In addition, the red color in the vitreous humor turned lighter 4 and 24 h after ANP‐HA‐GNP injection, supporting the notion of a proper diffusion capacity of the particles (Fig. 2G).

Analyzing the biodistribution of the ANP‐HA‐GNPs in retinal explants, administered particles were mainly observed in the photoreceptor layer of the retina (Fig. 2H). They were able to cross the inner limiting membrane and distributed from the ganglion cell layer to the photoreceptors. Nevertheless, there was a retention of ANP‐HA‐GNPs in the inner limiting membrane (Fig. 2H) due to the fact that the high particle volume used was not proportional to the explant size but was necessary to ascertain that the particles can penetrate and interact with the retinal tissue.

Taken together, (a) ANP‐HA were successfully attached to gold core nanoparticles, (b) a good uptake and intracellular distribution of the GNPS was overserved in ARPE‐19 cells and (c) good diffusion into the vitreous as well as *ex vivo* retinal biodistribution was verified. Thus, ANP‐HA‐GNPs have a promising potential to serve as antiangiogenic nanocarriers for the treatment of eye cancers like retinoblastoma.

### 3.3 *In ovo* tumor formation capacity of etoposide‐resistant RB cells after ANP‐HA‐GNP treatment

**Fig. 3:**
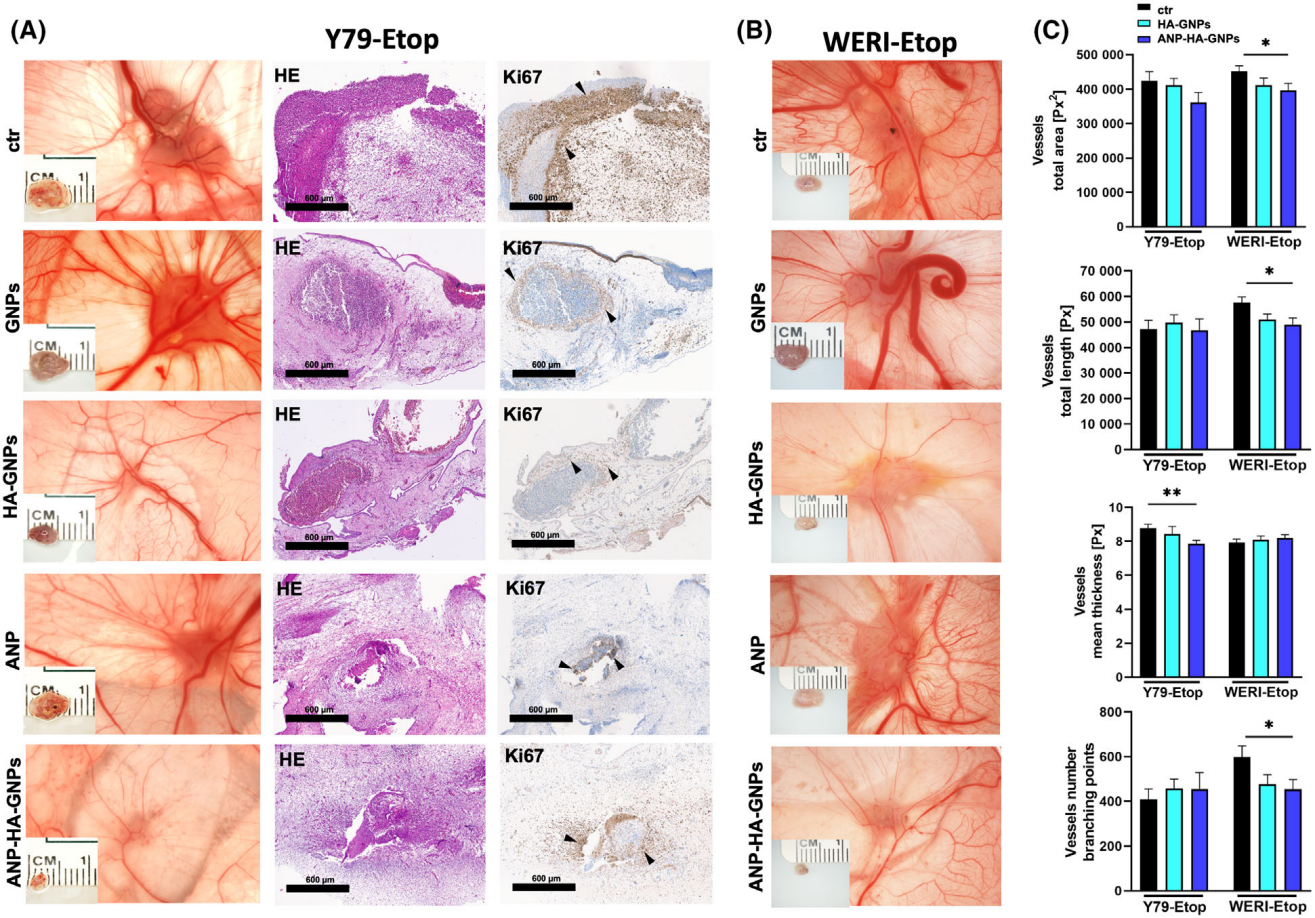
Effects of treatment with ANP coupled HA‐GNPs on tumor formation of etoposide resistant RB cells in *in ovo* chorioallantoic membrane (CAM) assays. Photographs of CAM tumors *in situ* and ruler measurements (in cm) of excised tumors revealing that tumors forming on the upper CAM 7 days after grafting of treated Y79‐Etop (A) and WERI‐Etop (B) cells were smaller compared to those arising from control cells treated with PBS (ctr). (A) Histological analysis of paraffin sections of Y79‐Etop CAM tumors by hematoxylin and eosin (HE) and Ki67 stains (brown signal). Black arrowheads exemplarily demarcate Ki67 positive cells positive cells. Scale bars: 600 μm. (C) Quantification of the total vessel area, vessel length, thickness and branching points of HA‐GNP and ANP‐HA‐GNP treated CAM tumors compared to the controls as calculated by an ikosa online software (KLM vision). The experiments were performed in triplicates. ANP, atrial natriuretic peptide; ANP‐HA‐GNP, ANP coupled HA‐GNPs; GNP, gold nanoparticles; HA‐GNP, hyaluronic acid coupled GNPs. Values represent means of independent animals ± SEM. *P < 0.05; **P < 0.01 statistical differences compared to the control group calculated by one‐way ANOVA with Newman–Keuls post‐test.

A previous study by our group revealed that etoposide resistant RB cells display a significantly increased tumor formation potential compared with chemotherapy‐sensitive cells of origin [39]. Thus, alternative treatment protocols are needed to reduce tumor growth of resistant RB cells. To investigate whether treatment with ANP‐coupled HA‐GNPs influences the tumor growth of etoposide‐resistant RB cells, we used the *in ovo* chicken chorioallantoic membrane (CAM) assay as a 3R‐conform prescreening model system to strengthen our hypothesis prior to treatment approaches in a classical rodent *in vivo* animal model. WERI‐Etop and Y79‐Etop cells were inoculated onto the CAM of 10‐day‐old chicken embryos and either treated with GNPs, HA‐GNPs, or ANP alone, conjugated ANP‐HA‐GNPs or PBS as a control. Photo‐documentation of CAM tumors developing from inoculated etoposide‐resistant Y79‐Etop (Fig. 3A) and WERI‐Etop cells (Fig. 3B) within 7 days showed a reduced tumor size after treatment with HA‐GNPs. This effect was strongly increased after treatment with ANP‐HA‐GNPs for both RB cell lines compared with the control cells treated with PBS. In addition, CAM tumors formed after inoculation of Y79‐Etop cells were analyzed immunohistochemically (Fig. 3A). Ki67 staining revealed proliferating tumor cells in each treatment group. In order to investigate the effect of the different treatments on vessel formation, we analyzed the total vessel area, vessel length, thickness, and branching points of PBS controls compared with HA‐GNP‐ and ANP‐HA‐GNP‐treated CAM tumors (Fig. 3C). We could show that treatment with HA‐GNPs alone had no effect on vessel formation. By contrast, in ANP‐HA‐GNP‐treated Y79‐Etop CAM tumors significantly reduced mean vessel thickness was observed and WERI‐Etop CAM tumors displayed a significantly reduced total vessel area, vessel length, and branching points.

**Fig. 4:**
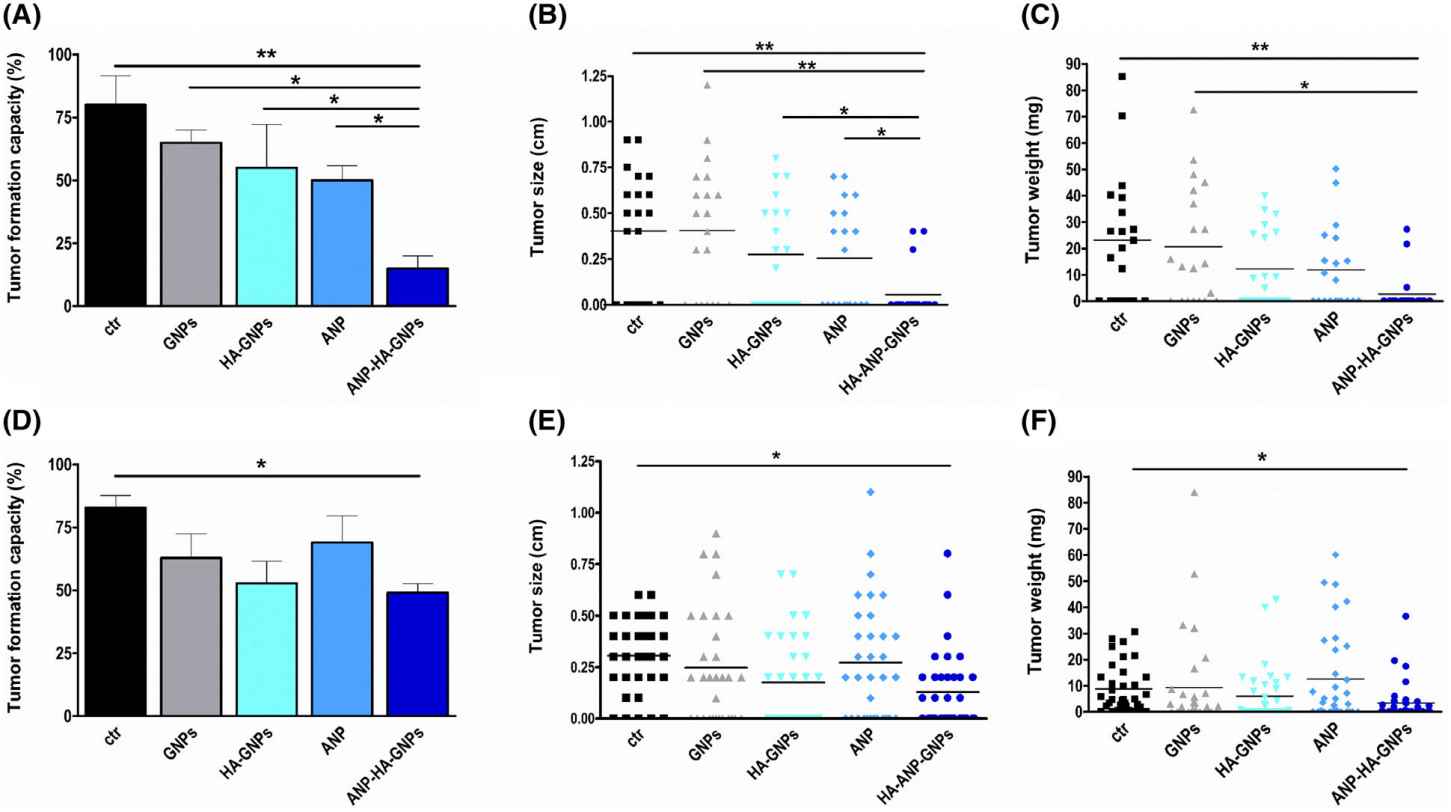
Quantification of tumor formation from grafted retinoblastoma (RB) cells after gold nanoparticle treatment in chorioallantoic membrane (CAM) assays. Tumor formation capacity (A, B), CAM tumor weight (B, E) and CAM tumor size (C, F) of etoposide resistant RB cells was quantified after treatment with GNPs, HA‐GNPs, ANP, or ANP‐HA‐GNPs. Upper row (A–C) shows the results for Y79‐Etop cells and lower row (D–F) data for WERI‐Etop cells. ANP, atrial natriuretic peptide; ANP‐HA‐GNP, ANP coupled HA‐GNPs; ctr, PBS treated; GNP, gold nanoparticles; HA‐GNP, hyaluronic acid coupled GNPs. Values are means of three independent experiments ± SEM. *P < 0.05; **P < 0.01 statistical differences compared to the control group calculated by one‐way ANOVA with Newman–Keuls post‐test.

Additionally, the tumor formation capacity of Y79‐Etop (Fig. 4A) and WERI‐Etop cells (Fig. 4D) was significantly reduced after treatment with ANP‐HA‐GNPs compared with control cells treated with PBS. Controls of the cell line Y79‐Etop also displayed a significantly decreased tumor formation capacity and tumor size in comparison with cells treated with GNPs, HA‐GNPs or ANP alone (Fig. 4A). Weight (Fig. 4B,E) and size (Fig. 4C,F) of CAM tumors developing from etoposide‐resistant RB cells treated with ANP‐HA‐GNPs were significantly lower compared with tumors forming from control cells treated with PBS only.

### 3.4 *In vivo* tumor formation of etoposide‐resistant RB cells in an orthotopic rat model

**Fig. 5:**
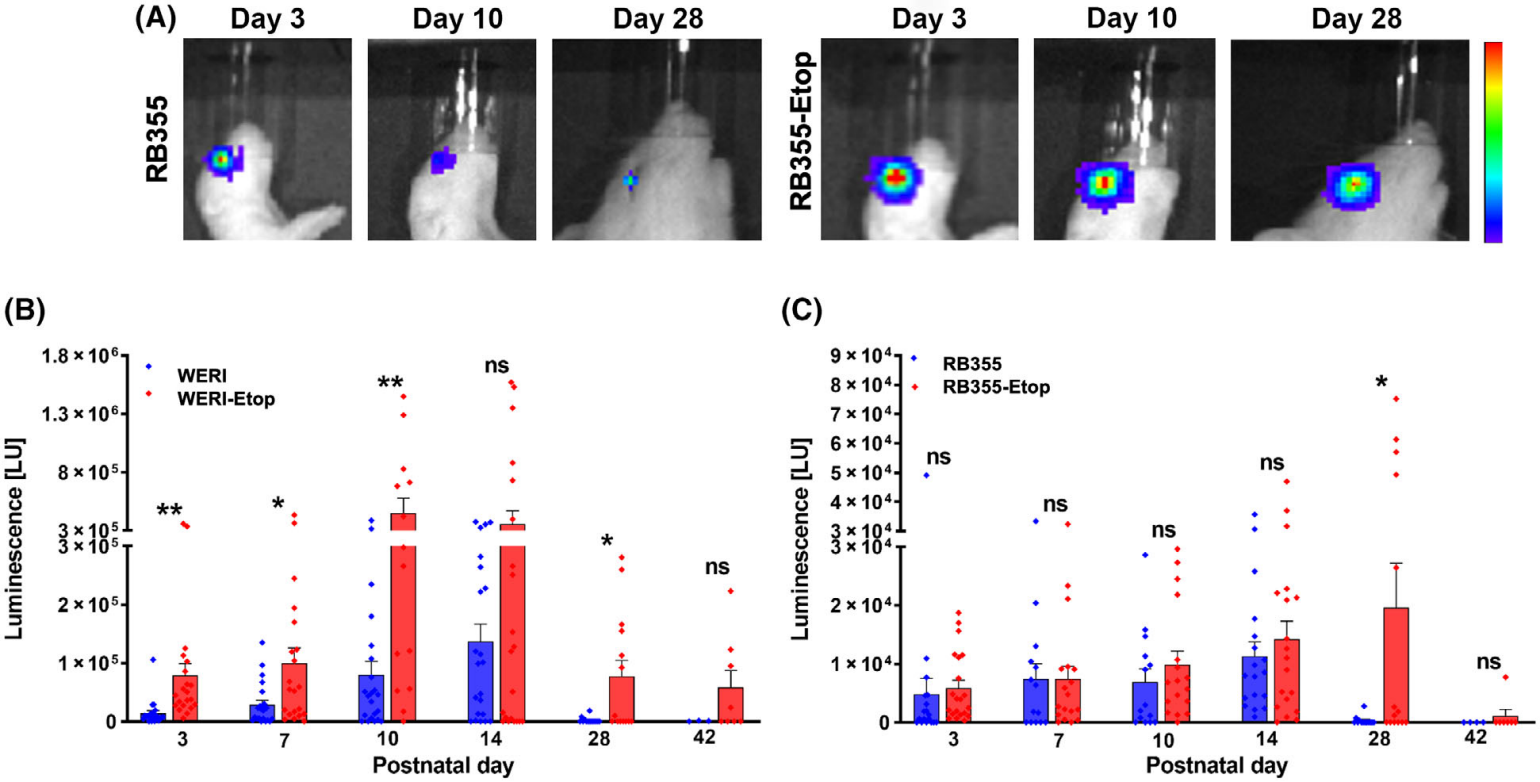
Luminescence signal (LU) of ocular tumors developing from chemosensitive and etoposide (−Etop) resistant WERI and RB355 RB cells injected into the eyes of newborn rats. (A) Pseudo‐color images of two representative animals injected with luciferase and green fluorescent protein (luc‐GFP) labeled sensitive and etoposide resistant RB355 cells at three time points (Day 3, 10, 28). Red color indicates highest luminescence intensity, dark blue color lowest luminescence signal. Quantification of luminescence signal measurements revealed increased tumor growth for WERI‐Etop (B) and RB355‐Etop (C) cells (red columns) compared with their chemosensitive counterparts (blue columns). Values are means of independent animals injected with WERI cells (*n* = 24), WERI‐Etop cells (*n* = 21), RB355 cells (*n* = 18), RB355‐Etop cells (*n* = 21), and phosphate buffered saline (PBS control; *n* = 3). Error bars indicate the SEM. ^ns^ *P* > 0.05; *P < 0.05; **P < 0.01 statistical differences compared to the control group (WERI or RB355) calculated by Student's *t*‐test.

Next, we set out to establish an orthotopic *in vivo* rat eye model for treatment of chemoresistant retinoblastoma tumors. To validate our *in vitro* and *in ovo* findings, suggesting that etoposide‐resistant RBs exhibit a more aggressive behavior than their chemosensitive cells of origin, we conducted *in vivo* investigations. We therefore injected luciferase and GFP‐labeled etoposide‐resistant RB cells (WERI‐Etop and RB355‐Etop) into the right eye of newborn rats 24 h after birth. Tumor growth was monitored by detection of the luciferase signal (Fig. 5A) over a time period of 9 weeks and compared with signal intensities of corresponding chemosensitive RB cell (WERI and RB355) tumors. We could show a high luciferase signal reflecting a high tumor formation capacity in at least 80% of the RB cell lines tested and observed a constant tumor growth until Day 14 postinjection (Fig. 5B,C). After 2 weeks, the luciferase signal dropped down in all RB cells investigated, except for RB355‐Etop cells (Fig. 5B,C). Nevertheless, we were able to verify our *in vitro* and *in ovo* data as WERI‐Etop cells showed a significantly increased tumor growth reflected by an increased luciferase signal compared with chemosensitive WERI cells until Day 28 (Fig. 5B) and RB355‐Etop cells displayed a slightly increased luciferase signal upon Day 14 reaching significance at Day 28 (Fig. 5C).

**Fig. 6:**
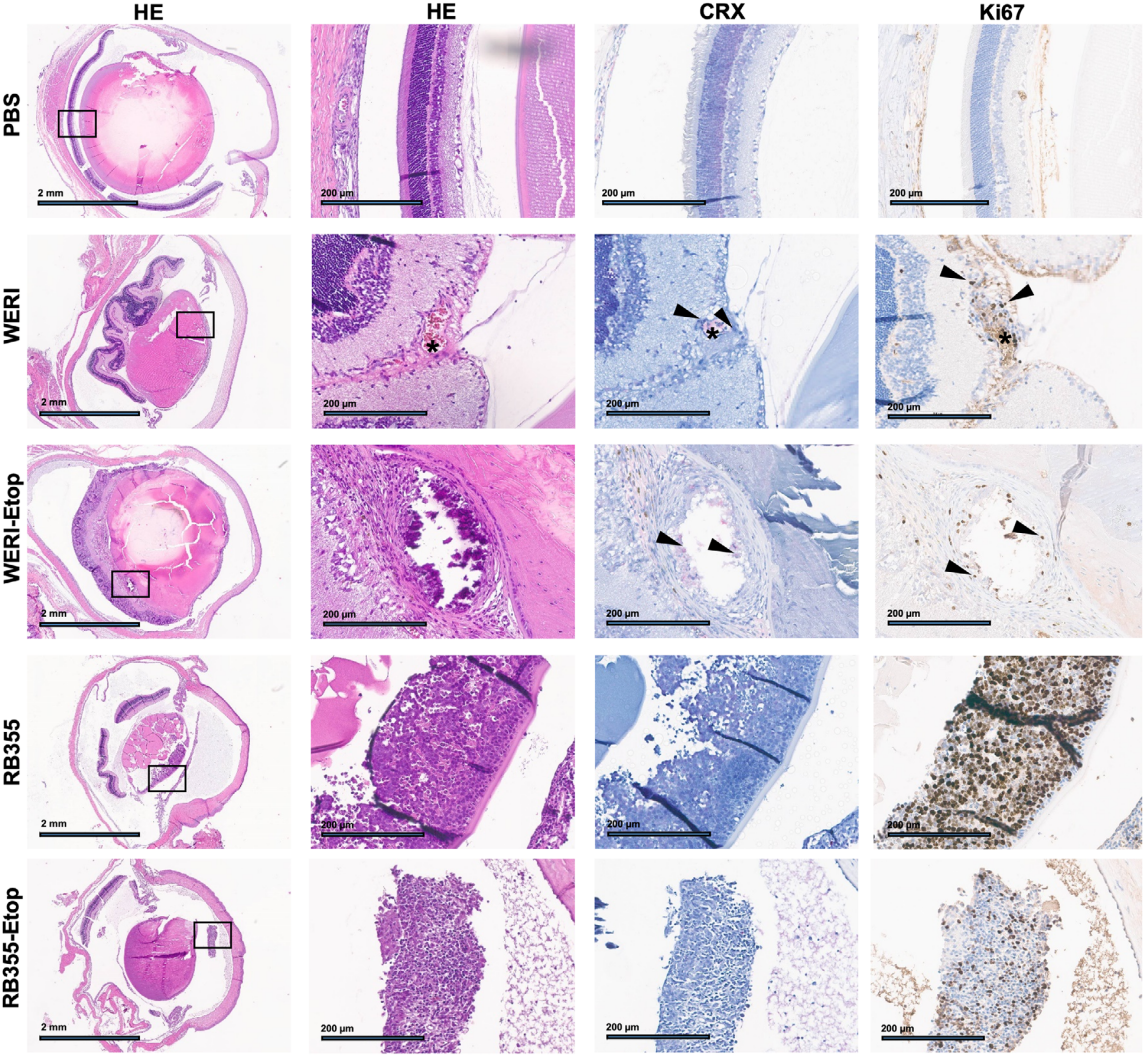
Histologic analysis of rat eye tumors after injection of human retinoblastoma (RB) cells. Paraffin sections of rat eyes 9 weeks after injection of chemosensitive and etoposide (−Etop)‐resistant WERI and RB355 RB cells revealed intravitreal tumors with CRX (light red signal) and Ki67 (brown signal) positive cells. PBS (upper row): control eye injected with PBS depicting normal retinal histology. HE: hematoxylin and eosin stains in two different magnifications (black boxes depict the zoom‐in area shown in the adjacent columns), Scale bars: 2 mm at 4× magnification (HE) and 200 μm in the zoom‐in area, CRX: retinal and RB marker, Ki67: proliferation marker, asterisk: blood vessel, arrowheads: RB tumor cells.

In order to analyze RB tumors grown *in vivo* histologically, enucleated rat eyes were embedded in paraffin, cut and immunohistologically stained for CRX and Ki67 to verify the RB origin and proliferation potential of the tumor xenografts. Hematoxylin and eosin stains proved tumor development for every RB cell line investigated (Fig. 6) supporting our luminescence‐based tumor data described above. In addition, we verified the RB origin of the tumors by CXR expression and were able to show proliferative activity of the tumor cells (Fig. 6). In the control eyes with PBS vehicle injection, no histological changes were visible upon 9 weeks of postinjection (Fig. 6).

Correlating with the observation that intraocular luciferase signals dropped down from Day 28 on, not all proven tumors were proliferative 9 weeks after injection. Therefore, experimental time line was shortened from 9 to 4 weeks for the following nanoparticle treatment approaches.

### 3.5 *In vivo* treatment effects of ANP‐HA‐GNPs on tumor formation of etoposide‐resistant RB cells in an orthotopic rat model

**Fig. 7:**
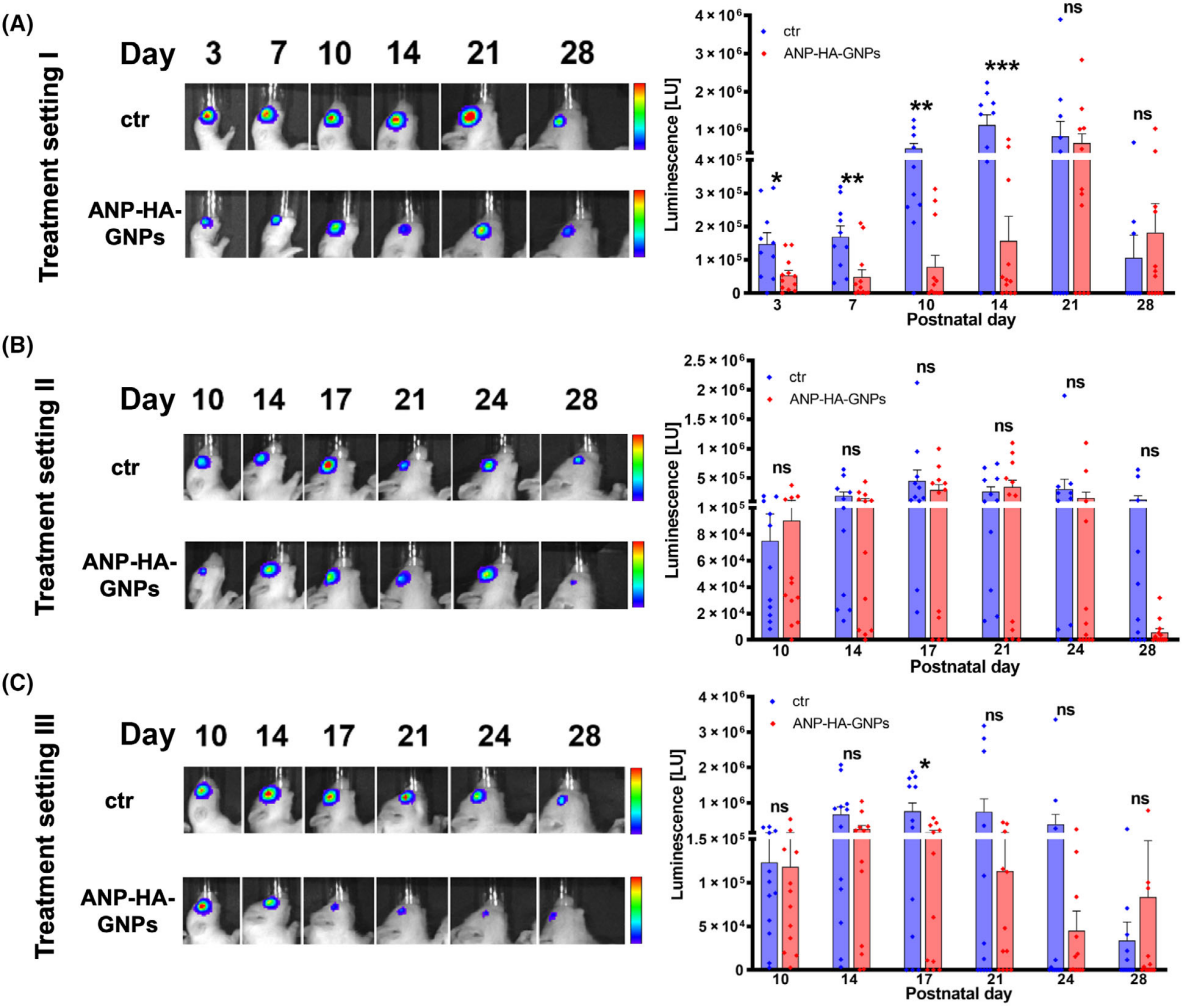
Luminescence signal (LU) of ocular tumors developing from etoposide (−Etop) resistant WERI RB cells injected into the eyes of newborn rats. (A) Treatment setting I: WERI‐Etop cells were injected into the right eye of P0 rat pups together with ANP‐HA‐GNPs (ctr: *n* = 10, ANP‐HA‐GNPs: *n* = 12). Treatment setting II and III: WERI‐Etop cells were allowed to form tumors 14 days prior to treatment with intravitreal injections of ANP‐HA‐GNPs (ctr: *n* = 11, ANP‐HA‐GNPs: *n* = 12; B) or topical treatment via eye drops (ctr: *n* = 12, ANP‐HA‐GNPs: *n* = 12; C). (A–C) Pseudo‐color images of representative animals injected with luciferase and green fluorescent protein (luc‐GFP) labeled etoposide resistant WERI cells at six different time points (Day 3–28; left side). Red color indicates highest luminescence intensity, dark blue color lowest luminescence signal. Quantification of luminescence signal measurements (right side) revealed differences in tumor growth between ANP‐HA‐GNPs treated WERI‐Etop cells (red columns) and untreated WERI‐Etop control cells (blue columns). Values represent means of independent animals ± SEM; significances were calculated by unpaired Student's *t*‐test. ^ns^ *P* > 0.05; *P < 0.05; **P < 0.01; ***P < 0.001.

We set out to test the effectiveness and potential application routes of ANP‐HA‐GNPs to treat etoposide‐resistant RB cells *in vivo*. As a proof of principle and to verify the *in ovo* effects of ANP‐HA‐GNPs on RB tumor growth, we injected WERI‐Etop cells together with ANP‐HA‐GNPs into the rat eyes 24 h after birth (treatment setting I). We could show that in comparison with controls tumor growth of the treated WERI‐Etop cells were significantly reduced 14 days upon injection (Fig. 7A). In treatment setting II and III, more closely resembling the actual conditions in RB patients, we let the tumor grow for 2 weeks prior to treatment with ANP‐HA‐GNPs. Thereupon, in treatment setting II, we injected the ANP‐HA‐GNPs into the rat eyes with RB tumor growth and used ANP‐HA‐GNP eye drops as topical treatment under treatment regimen III. As shown in Fig. 7B, the injection regimen of ANP‐HA‐GNPs in treatment setting II did not significantly reduce RB tumor growth in comparison to the controls, whereas topical administration of ANP‐HA‐GNPs eye drops led to a significant tumor reduction after the first treatment cycle at Day 17 and further, not yet significant, reduced tumor growth upon Day 24 (Fig. 7C).

**Fig. 8:**
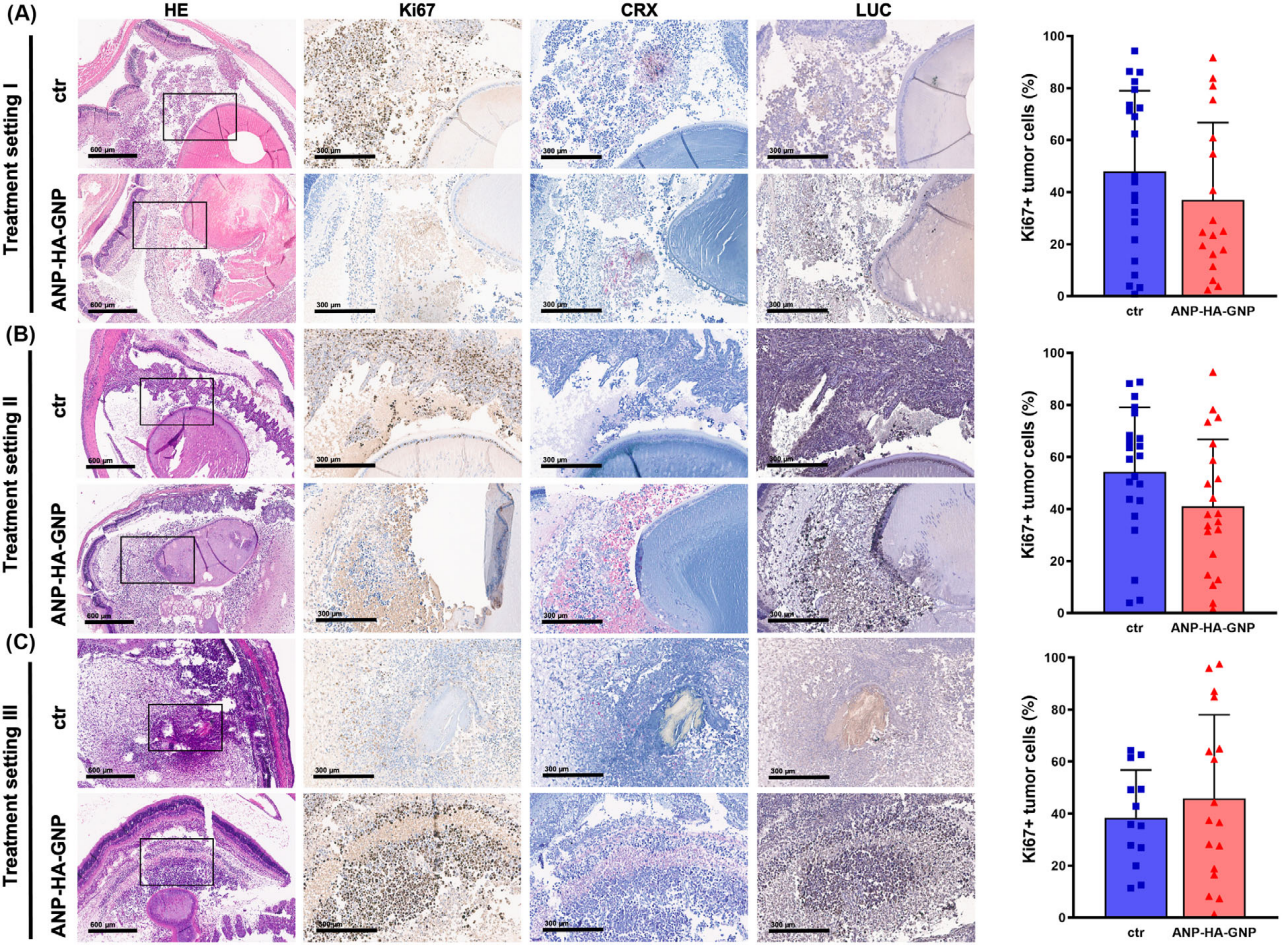
Histological analysis of rat eye tumors after injection of human retinoblastoma (RB) cells and treatment with gold nanoparticles. Paraffin sections of rat eyes 4 weeks after different treatments setting. (A) Treatment setting I: Etoposide (−Etop)‐resistant WERI‐Etop cells were injected into rat pups eyes together with ANP‐HA‐GNPs (ctr: *n* = 10, ANP‐HA‐GNPs: *n* = 12). Treatment setting II and III: WERI‐Etop cells were allowed to form tumors prior to treatment with intravitreal injections of ANP‐HA‐GNPs (ctr: *n* = 11, ANP‐HA‐GNPs: *n* = 12; (B) or topical treatment via eye drops (ctr: *n* = 12, ANP‐HA‐GNPs: *n* = 12; (C). Intravitreal tumors with CRX (light red signal), Ki67 (brown signal) and luciferase (LUC, brown signal)) positive cells. ctr (upper rows): control eyes treated with PBS, HE: Hematoxylin and eosin stains (black boxes depict the zoom‐in area shown in the adjacent columns), CRX: retinal and RB marker, Ki67: proliferation marker, LUC: luciferase expressing cells, Scale bars: 600 μm at 4× magnification and 300 μm at 10× magnification Ki67 positive (Ki67+), proliferating tumor cells were quantified using imagej. ctr: PBS treated (blue bars). ANP, atrial natriuretic peptide; ANP‐HA‐GNP, ANP coupled HA‐GNPs (red bars); GNP, gold nanoparticles; HA, hyaluronic acid. Values represent means of independent animals ± SEM.

After 4 weeks of RB tumor growth, we enucleated the rats eyes of all three treatment regimen and performed histological stainings for Ki67, CRX, and luciferase to verify the RB origin and the proliferation potential of the tumor xenografts (Fig. 8). Hematoxylin and eosin as well as luciferase stains revealed RB tumor development, while CRX positivity proved their RB tumor cell origin and Ki67 staining verified a positive proliferation status of the tumor. The majority of the RB tumors were positive for all markers analyzed. In treatment setting I and II, ANP‐HA‐GNP‐treated tumors showed a slightly reduced proliferation rate with less Ki67‐positive RB tumor cells in comparison with the control group. This reduction did, however, not reach significance (Fig. 8).

In conclusion, we verified that ANP‐HA‐GNPs inhibit tumor growth *in vivo*, but a suitable treatment modality and the most effective treatment cycle need to be further elaborated. Generally, treatment with eye drops displayed to be effective in reducing tumor growth, but it seems that the dose needs to be increased, for example, by more frequent administrations of the ANP‐HA‐GNPs.

## 4 Discussion

Retinoblastoma is an ophthalmological childhood cancer with serious consequences if left untreated, for example, loss of vision, secondary cancers, and death [7, 41]. Systemic or local chemotherapy treatment regimens effectively reduce tumor size, inhibit metastasis, and preserve vision [5, 42, 43]. Chemotherapy can, however, cause the development of resistant RB tumor cells. We recently demonstrated that compared with corresponding chemosensitive cells of origin etoposide‐resistant RB cells are more aggressive in terms of increased proliferation and tumor formation *in ovo* [39]. Therefore, new additional treatment approaches are needed to either prevent tumor growth from residual resistant RB cells developing upon chemotherapy or even treat resistant RB cells after recurrence. Nowadays, the development of new diagnostic and treatment approaches, also in the context of retinoblastoma, focusses on multifunctionalized nanocarriers (for review see Ref. [44]). These nanocarriers can be used to effectively transport drugs, peptides, or nucleic acids to tumor sites [45]. For RB, feasible ocular application routes comprise systemic, topical, periocular, intravitreal, and suprachoroidal approaches [46]. A noninvasive topical administration via eye drops would be the most convenient and desirable treatment option for children. Up to now all nanocarrier systems including organic polymers and inorganic nanoparticles loaded with different drugs displayed an increased bioavailability and reduced side effects compared with application of coupled anticancer agents alone [44].

Inorganic gold nanoparticles (GNPs) have naturally antioxidant and antiangiogenic properties [8] and the potential to reduce proliferation and migration of retinal pigment epithelium (RPE) cells [9]. Besides, most recent studies demonstrated that GNPs can either be combined with ultrasonic hypothermia [47] or laser therapy [48] to enhance RB cell death. Small sized GNPs are able to pass anatomical barriers of the eye [12], a property augmented by combining a gold core with a hyaluronic acid (HA) coat, allowing for binding to the CD44 surface receptor expressed by several cells of the eye [11, 13, 15]. We recently demonstrated an increased mobility and permeability of HA‐GNPs through ocular barriers resulting in antiangiogenic effects via inhibition of neovascularization [11]. These previous data are in good accordance with effects seen in the study presented, in which HA‐GNP treatment of etoposide‐resistant cells reduced RB tumor growth in an *in ovo* CAM assay.

It has been demonstrated that the atrial natriuretic peptide (ANP) reduces choroidal neovascularization by inhibiting the vascular endothelial growth factor (VEGF) [19], shown to be expressed in RB and to correlate with increased malignancy [20]. Besides, combined treatment with glipizide and ANP not only inhibited angiogenesis but also effectively suppressed breast cancer growth and metastasis via the VEGFR signaling axis [22]. Against this background, we set out to attach ANP to HA‐GNPs in order to create a nanocarrier combining two properties: a high ocular delivery rate due to a HA coat and antitumorigenic qualities due to attached ANP to effectively reduce RB tumor growth. In the study presented, we successfully coupled ANP to HA‐GNPs and showed that these nanoparticles are internalized by ARPE‐19 cells and display a homogenous distribution. In addition, an efficient diffusion of ANP‐HA‐GNPs into the vitreous humor of *ex vivo* retina explants was observed, and the nanoparticles were able to cross the inner limiting membrane and ganglion cell layer of the retina to reach the photoreceptors. Thus, the basic conditions for treatment applications were fulfilled. Next, testing antitumorigenic and antiangiogenic effects of the GNPs on etoposide‐resistant RB cells *in ovo*, we revealed that the tumor‐repressive effect of HA‐GNPs treatment on RB tumor growth significantly increased upon coupling of ANP to the nanoparticles. Besides, it significantly affected vessel development. These results are in good accordance with previous studies showing that ANP induces various antitumor effects in different cancer entities (for review see Ref. [49]). Moreover, an ANP‐derived peptide (KTH‐22) inhibited pancreatic cancer cells more effectively than gemcitabine [50]. ANP mainly signals through two specific plasma membrane receptors, the natriuretic peptide receptor A (NPRA) and the natriuretic peptide receptor C (NPRC), and modulates expression and signaling of different molecules including the Ras‐MEK1/2‐ERK1/2 kinase cascade, the Wnt‐β‐catenin pathway, VEGF, and JNK/JAK/STAT signaling, ultimately leading to anticancer effects [49, 51].

Based on previous published work by Corson et al. [34], in the study presented, we established an orthotopic rat eye model system to test different RB treatment approaches in an ocular *in vivo* situation. In this model, we observed significantly increased tumor growth of etoposide‐resistant WERI and RB355 cells *in vivo*, verifying our previous *in vitro* findings that etoposide‐resistant RB cells behave more aggressively compared to their chemosensitive counterparts [39]. Besides, we investigated three different application routes for the ANP‐HA‐GNPs in order to find the most effective ocular drug delivery route to reduce RB tumor growth. As a proof of principle, we injected etoposide‐resistant WERI cells together with ANP‐HA‐GNPs into newborn rat eyes and observed significantly decreased tumor growth 14 days after treatment. To investigate the properties of ANP‐HA‐GNPs in a more clinical setting, we treated already developed RB tumors in rat eyes via injection or topical administration of nanoparticles via eye drops. Topical administration reduced tumor growth of resistant RB cells, whereas the injection therapy approach did not change the tumor formation capacity compared with control cells. Thus, the effect of ANP‐HA‐GNP eye drops is promising, nevertheless, should be optimized by increased dose rates and/or more frequent administrations.

Next to intracellular effects on several signaling molecules described above, ANP has been shown to modulate inflammation, a hallmark of cancer known to promote tumor progression, metastasis and drug resistance [52, 53]. Along this line, it could be demonstrated that lung cancer patients treated with ANPs had longer 2‐year relapse‐free survival time [54] and reduced inflammatory responses [55, 56]. These effects are possibly trigged via the ANP‐NPRA signaling axis [54], which also plays a role in tumor–stroma interactions [57], rendering it a potential therapy target in the context of inflammation‐associated tumorigenesis [49]. Interestingly, previous studies also showed prophylactic effects on recurrence of lung cancer after ANP therapy [49] and protection from cisplatin induced renal dysfunction and renal tubular necrosis, a major toxicity after cisplatin therapy [58]. Thus, ANP is a highly attractive candidate for future cancer therapies as it mediates antiproliferative and anti‐inflammatory effects and offers the potential to circumvent cytotoxic side effects of conventionally used chemotherapeutics such as cisplatin, also used in RB therapy.

Taken together, we established functionally active GNPs with a hyaluronic acid coat leading to increased ocular accessibility. Additional coupling of ANP to these effective nanocarriers reduced angiogenesis and further increased the antitumorigenic effect on resistant RB cells *in vivo*. Therefore, ANP‐HA‐GNPs are a promising new adjuvant therapy option to treat RB tumors via non‐invasive eye drops and/or for use as prophylactic agents to prevent recurrence upon the development of chemoresistance. Nevertheless, downstream signaling effectors of the ANP‐HA‐GNPs need to be investigated in more detail in future experiments.

## 5 Conclusion

In the study presented, we demonstrated that compared with parental chemosensitive tumor cells etoposide‐resistant RB tumor cells exhibit a more aggressive growth behavior in an established *in vivo* orthotopic rat model. To treat these resistant cells, we established ANP‐coupled, HA‐coated gold nanoparticles, which displayed a good ocular biodistribution. *In ovo* CAM experiments demonstrated for the first time a RB tumor reducing effect of ANP‐HA GNPs, which was confirmed in the *in vivo* orthotopic rat model. To identify an optimal application route for a potential future clinical RB therapy approach, ANP‐HA GNPs were injected into RB tumor bearing rat eyes as well as administered by eye drops. The less invasive treatment method via eye drops seems to be the most effective administration strategy, however, this finding needs to be further evaluated. Overall, we demonstrated that the synthesized ANP‐HA GNPs effectively reduce RB tumor growth *in ovo* and *in vivo* and administered as eye drops might potentially serve as a useful adjunct to standard RB therapy.

## Conflict of interest

The authors declare no conflict of interest.

## Author contributions

MAB and PSA conceptualized the study. AH, NM, SK, AD, DVM, AJ, AG, and PSA performed the methodology. AH, NM, SK, AD, PSA, and MAB involved in investigation. MAB, PSA, and ND curated the data. MAB, PSA, ND, and AH wrote the original draft preparation. MAB and ND reviewed, and edited the manuscript. MAB, PSA, AH, and NM visualized the data. MAB supervised the study. ND and AG involved in project administration. All authors have read and agreed to the published version of the manuscript.

## Supporting information


**Fig. S1.** Transmission electron microscopy (TEM) of a retinoblastoma (RB) cell after gold nanoparticle uptake.

## Data Availability

The data that support the findings of this study are available from the corresponding author (maike.busch@uk-essen.de) upon reasonable request.
