# Supplementary material for: New retinoblastoma (RB) drug delivery approaches: anti‐tumor effect of atrial natriuretic peptide (ANP)‐conjugated hyaluronic‐acid‐coated gold nanoparticles for intraocular treatment of chemoresistant RB
Source: Mol Oncol. 2024 Jan 12;18(4):832–49. doi: 10.1002/1878-0261.13587 (PMC10994242; doi:10.1002/1878-0261.13587)
Supplement: Supplementary file 1 — Fig. S1. Transmission electron microscopy (TEM) of a retinoblastoma (RB) cell after gold nanoparticle uptake. [file MOL2-18-832-s001.zip › Supplementary Figure legend.docx]

**Supplementary Figure 1.** Transmission electron microscopy (TEM) of a retinoblastoma (RB) cell after gold nanoparticle uptake. TEM microscopy pictures of internalized ANP-HA-GNPs (white box in A,B; shown at higher magnification in C) in a Weri-Etop RB cell. Photos were taken 48 h after treatment with ANP-HA-GNPs. (A) Scale bar: 2.5 µm at 3.000x magnification. (B) Scale bar: 250 nm at 20.000x magnification. (C) Scale bar: 100 nm at 80.000x magnification. ANP: atrial natriuretic peptide; HA: hyaluronic acid; GNP: gold nanoparticles; ANP-HA-GNP: ANP coupled HA-GNPs
