# Supplementary figures and images for: New retinoblastoma (RB) drug delivery approaches: anti‐tumor effect of atrial natriuretic peptide (ANP)‐conjugated hyaluronic‐acid‐coated gold nanoparticles for intraocular treatment of chemoresistant RB
Source: Mol Oncol. 2024 Jan 12;18(4):832–49. doi: 10.1002/1878-0261.13587 (PMC10994242; doi:10.1002/1878-0261.13587)

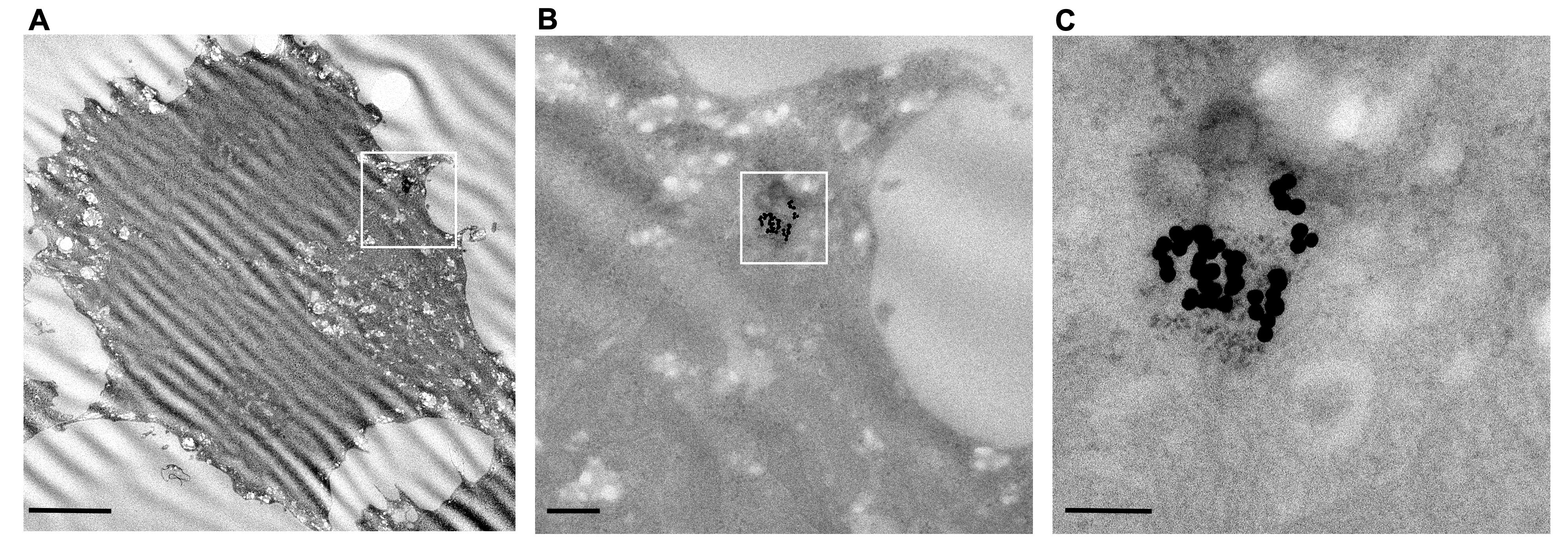

Supplement: Supplementary file 1 — Fig. S1. Transmission electron microscopy (TEM) of a retinoblastoma (RB) cell after gold nanoparticle uptake. [file MOL2-18-832-s001.zip › Supplementary Figure 1.tiff]
